# Validation of a Three-Dimensional Computed Tomography Reconstruction Tool for Aortic Valve Calcium Quantification

**DOI:** 10.1016/j.shj.2022.100122

**Published:** 2022-11-28

**Authors:** Thijmen W. Hokken, Joris F. Ooms, Isabella Kardys, Alexander Hirsch, Annick C. Weustink, Sanne Schipper, Peter Heil, Joost Daemen, Ricardo P.J. Budde, Nicolas M. Van Mieghem

**Affiliations:** a Department of Cardiology, Thoraxcenter, Erasmus University Medical Center, Rotterdam, the Netherlands; b Department of Radiology and Nuclear Medicine, Erasmus University Medical Center, Rotterdam, the Netherlands; c Pie Medical Imaging, Maastricht, the Netherlands; d 3mensio Medical Imaging, Bilthoven, the Netherlands

**Keywords:** Agatston score, Aortic valve calcification, Aortic valve stenosis

## Abstract

**Background:**

Aortic valve calcification correlates with the severity of aortic valve stenosis and a high calcium score is associated with conduction disturbances and paravalvular leakage after transcatheter aortic valve replacement. The 3mensio Structural Heart is a semiautomated software package to facilitate aortic root analysis by multislice computed tomography.

The aim of the contemporary study is to validate a semiautomated calcium quantification scoring tool with a conventional manual calcium quantification tool.

**Methods:**

Fifty randomly selected patients who underwent multislice computed tomography for preprocedural planning were retrospectively selected to compare the semiautomated aortic valve Agatston calcium score by 3mensio with the manually obtained score using IntelliSpace Portal as standard reference.

**Results:**

Patients had a mean age of 76.7 ± 7.4 years and 60% were male. The median Agatston score was 3390 [interquartile range 1877-4509] with 3mensio and 3434 [interquartile range 1839-4620] with IntelliSpace.

The mean difference was -0.18 [95% confidence interval (CI) −53.8 to 53.4]. The intraclass correlation coefficient between the Agatston scores using IntelliSpace and 3mensio showed an excellent correlation of 0.995 [95% CI 0.992-0.997], *p* ≤ 0.001. The interobserver and intraobserver variability was 0.993 ([95% CI 0.961-0.998], *p* ≤ 0.001) and 0.995([95%CI 0.981-0.999], *p* = <0.001), respectively.

**Conclusions:**

The semiautomated calcium quantification module in 3mensio Structural Heart highly correlated with a conventional manual calcium scoring tool.

## Highlights

• Aortic valve calcification is a predictor for aortic stenosis severity and for adverse outcomes after TAVR.
• The semiautomated Agatston score module in 3mensio highly correlated with a conventional manual calcium scoring tool.
• The semiautomated Agatston score module in 3mensio makes addition and quantification of the calcium distribution to the preprocedural planning accessible.

## Introduction

Aortic valve stenosis (AS) is the most common valvular heart disease. The prevalence increases by age, affecting around 3.4% of elderly patients >75 years.^1^ In the Western world, degenerative aortic valve disease, characterized by the presence of aortic valve calcification (AVC) and associated with valve stiffening and thickening, is the predominant cause of AS.^2^ AVC shares similar risk factors to those for coronary atherosclerosis, including age, hypertension, smoking, and hyperlipidemia.^3^ The amount of calcification correlates with the severity of aortic stenosis.^4^ The Agatston score is a calcium quantification score which initially was used for determination of the quantity of coronary calcium on non-contrast enhanced scans.^5^ This Agatston score has also been validated to quantify AVC and gender-specific thresholds have been reported for severe AS. The European guidelines stipulate that an Agatston score >2000 for men and >1200 for women denotes severe AS.^6^

Transcatheter aortic valve replacement (TAVR) is an alternative to surgical aortic valve replacement.^6^ Multislice computed tomography (MSCT) is key for preprocedural TAVR planning, patient selection, transcatheter heart valve sizing based on aortic root dimensions and phenotype and access strategy.^7^ MSCT also allows to assess aortic root calcium burden and distribution. A high amount of AVC is a risk factor for cardiovascular events, conduction disturbances, and paravalvular leakage after TAVR.^8^, ^9^, ^10^

The 3mensio Structural Heart (Pie Medical Imaging, Maastricht, the Netherlands) is a dedicated software package for semiautomated MSCT analysis and shows adequate correlation with manual MSCT analysis in terms of annular and arterial assessments.^11^^,^^12^ The software package that so far included a semiautomated calcium volume quantification now introduces a novel semiautomated calcium quantification module expressed as an Agatston score. Herein, we aimed to validate the novel 3mensio calcium scoring tool by comparing the Agatston score obtained by 3mensio with the Agatston score using a conventional manual CT workstation.

## Methods

A total of 50 randomly selected patients who underwent preprocedural MSCT planning for TAVR between January 2017 and January 2021 were retrospectively selected for this validation study. Contrast-enhanced cardiac MSCT studies were performed on a dual source CT scanner (SOMATOM Force, Siemens Healthcare, Forchheim, Germany). Imaging included an electrocardiography (ECG)-gated non-contrast scan, at a time delay of 280 ​ms after the R-peak with slice thickness of 3mm and an ECG-gated contrast enhanced scan with multiple phases reconstructed during systole (at every 5% between 20% and 50% of the R-R interval).

MSCT-images were analyzed by experienced radiologists using a commercially available standard CT workstation (IntelliSpace Portal, Philips Healthcare, Amsterdam, the Netherlands). The calcium scoring application in the IntelliSpace Portal automatically displays all pixels with a Hounsfield unit (HU) value of 130 or higher. Calcium has to be selected manually on the axial images. A region growing algorithm extends the selected region of interest to all connected pixels with a HU value of ≥130.

The region of interest to determine AVC included the aortic valve leaflets and the (virtual) annulus defined by the leaflet hinge points. Calcifications in the aortic sinus, coronary arteries, left ventricular outflow tract, and mitral annulus were excluded. If a selected calcification in the aortic valve was in direct contact with calcifications outside the aortic valve (e.g., the mitral valve annulus) the region growing algorithm was not used. All calcifications deemed to be within the aortic valve were outlined manually in each slice. Calcium was defined by a fixed attenuation threshold of a minimum of 130 HU. The density score was determined as followed: a weighting factor of 1 was assigned for an area of 130-199HU, a factor 2 for an area of 200-299HU, factor 3 for an area of 300-399HU, and factor 4 for an area >400HU. The density score was multiplied by the area and the sum of all weighted areas in the region of interest generated the total Agatston score.

The same MSCT-images were then analyzed with the 3mensio Structural Heart. A detailed description has been published earlier.^11^^,^^12^ Briefly, the aortic valve and root were automatically reconstructed from the ECG-gated contrast scan at 30% of the RR-interval. The annulus was manually traced by selecting the nadir of the 3 cusps. After conformation of the aortic annulus, the non-contrast ECG-gated scan was selected for calcium quantification. The module automatically copies the previous aortic root 3-dimensional reconstruction in the non-contrast scan. Areas with ≥130HU between 5.0mm below and 20mm above the aortic annulus are automatically selected. Fine-tuning of calcium identification and delineation of the region of interest could require (minimal) manual adjustments.

All analyses were performed independently and blinded from each other. The study was conducted in accordance with the Declaration of Helsinki and did not fall under the scope of the Medical Research Involving Human Subjects Act per institutional review boards’ review.

### Statistical Analysis

Distributions of continuous variables were tested for normality with the Shapiro-Wilk test. Continuous variables were reported as mean ± standard deviation if normally distributed or as median (25th-75th percentile) if non normally distributed. Categorical variables were reported as number and percentage. A Bland-Altman plot was used to asses agreement between the 2 Agatston scores. The intraclass correlation coefficient (ICC) was calculated for the 2 modalities. Moreover, 10 randomly selected cases were independently analyzed by 2 imagers to obtain interobserver and intraobserver variability by calculating an ICC. All statistics were performed with SPSS software version 25.0 (SPSS, Chicago IL, United States).

## Results

### Study Population

Patient characteristics, including medical history and CT-data are shown in Table 1. The mean age was 76.7 ± 7.4 years and 60% were male. The mean annulus area was 459.9 ± 87.8mm2 with a mean diameter of 24.3 ± 2.3mm. The ostium of the left coronary artery was at a mean height of 14.0 ± 2.9mm and of the right coronary artery at 18.2 ± 2.9mm.

**Table 1:** Study population characteristics

Study population characteristics	Total population (N = 50)
Male	30 (60)
Age	76.7 ± 7.4
BMI (kg/m^2^)	27.2 ± 4.6
Medical history	
Hypertension	32 (64)
Hypercholesterolemia	33 (66)
Diabetes	11 (22)
Peripheral vascular disease	14 (28)
History myocardial infarction	2 (4)
History PCI	7 (14)
History CABG	2 (4)
Stroke	10 (20)
COPD	7 (14)
Renal failure	12 (24)
Pacemaker at baseline	0
CT analysis	
Annulus area (mm^2^)	459.9 ± 87.7
Annulus mean diameter (mm)	24.3 ± 2.3
LVOT area (mm^2^)	435.3 ± 94.7
LVOT mean diameter (mm)	23.4 ± 2.5
SOV area (mm^2^)	873.7 ± 211.1
Right coronary height (mm)	18.2 ± 2.9
Left coronary height (mm)	14.0 ± 2.9

Data are presented as mean ± standard deviation or number (percentage).

BMI, body mass index; CABG, coronary artery bypass grafting; COPD, chronic obstructive pulmonary disease; CT, computed tomography; LVOT, left ventricular outflow tract; PCI, percutaneous coronary intervention; SOV, Sinus of Valsalva.

### Validation of the Calcium Quantification Tool

**Figure 1:**
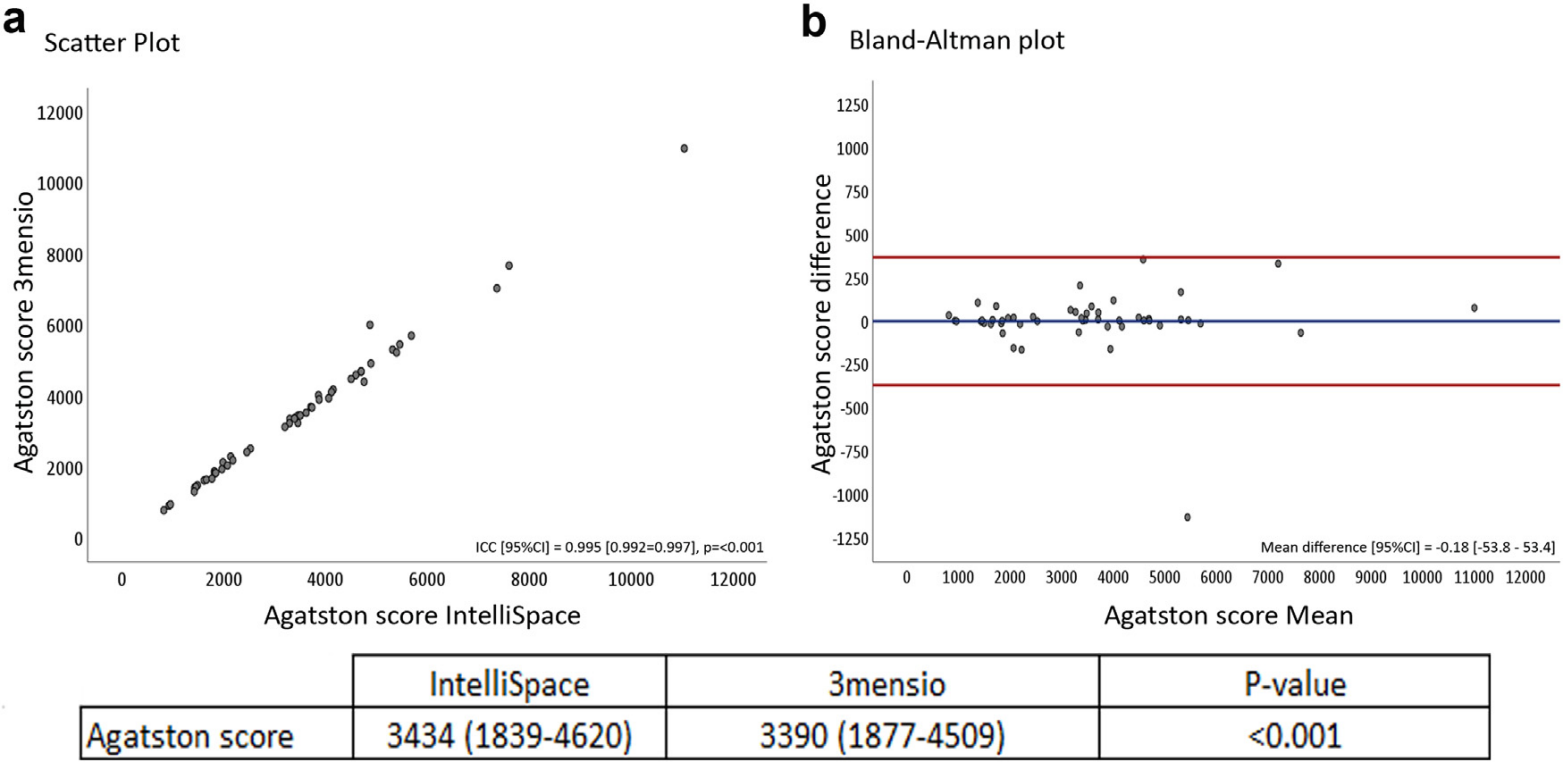
Scatter plot and Bland-Altman plot, A: Scatter plot of Agatston score determined by IntelliSpace and 3mensio with the calculated ICC [95% CI]. B shows the Bland-Altman plot with the mean difference. Abbreviations: ICC, intraclass correlation coeficient; CI, confidence interval.

The median (25th-75th percentile) Agatston score through manual analysis on the IntelliSpace workstation was 3434 (1839-4620) and using the semiautomated 3mensio Heart Valve was 3390 (1877-4509), *p* < 0.001. The mean difference was -0.18 [95% confidence interval (CI) −53.8 to 53.4]. In 5 patients (10%), the difference between the 2 Agatston scores was >5% and in only 1 patient the difference was >10%. Figure 1 shows the Bland-Altman plot. The ICC between the Agatston scores determined using the 2 image modalities showed a correlation of 0.995 [95% CI 0.992-0.997], *p* ≤ 0.001. The interobserver and intraobserver ICC were 0.993 [95% CI 0.961-0.998], *p* ≤ 0.001 and 0.995 [95% CI 0.981-0.999], *p* <0.001, respectively.

## Discussion

In this validation study, we reported an excellent correlation for aortic calcification quantification by Agatston score between the novel semiautomated 3mensio Structural Heart package and the conventional IntelliSpace manual CT workstation.

Assessment of AVC becomes increasingly important for patients with AS. First, the aortic valve Agatston score is incorporated in contemporary guidelines to further quantify aortic stenosis severity, especially in patients with low-flow, low-gradient AS.^6^ Distribution of calcium is also an important predictor for outcomes after TAVR. High calcium load, especially in the left coronary cusp, is associated with an increased risk for conduction disturbances, including new pacemaker implantation, after TAVR.^13^ AVC is also associated with asymmetrical transcatheter valve prosthesis expansion, which may result in paravalvular leakage that often is located around the calcified areas.^8^ In aggregate, calcium quantification is an essential component of preprocedural planning for TAVR.

**Figure 2:**
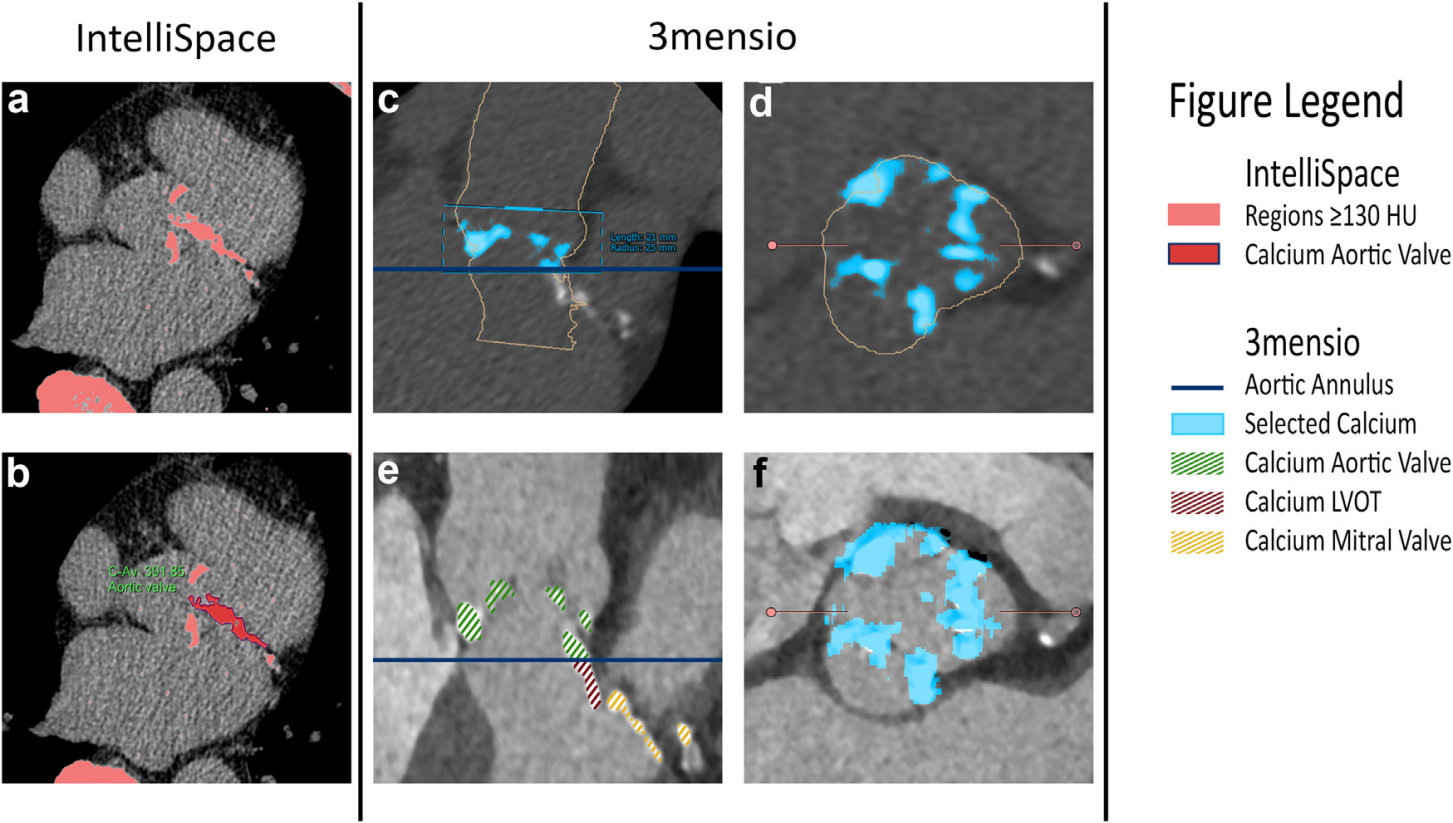
Example of the same calcified lesions in 3mensio and IntelliSpace. Figure A and B are the calcium determination in IntelliSpace. In A, all calcium with a HU ≥ 130 has been selected. B shows the manual selection of the aortic valve calcium. Figure C to F is the 3mensio calcium scoring module. C (double oblique view) and D (perpendicular plane) are the non-contrast scan in 3mensio with the selected aortic valve calcium in blue. E is the double oblique contrast scan which makes the calcified lesions of the aortic valve, LVOT and mitral valve visible, and F is the perpendicular plane of the contrast scan. Abbreviations: HU, Hounsfield units, LVOT, left ventricular outflow tract.

3mensio Structural Heart is a dedicated program that has been widely used during the preprocedural planning for determination of the aortic valve dimensions and for access site management. So far, only calcium volume quantification was possible with this software to The addition of the calcium Agatston quantification module refines calcium scoring and provides an additional advantage with the option to calculate the calcium burden per leaflet. The contemporary study showed an excellent correlation with the manual Agatston scoring module in IntelliSpace (ICC 0.995 [95% CI 0.992-0.997], *p* ≤ 0.001). The semiautomated Agatston scoring module is the first software tool that involves a combination of the ECG-gated contrast enhanced scan with 0.6mm thick slices and the non-contrast scan. The combination of the reconstructions using contrast and non-contrast scans may simplify the determination of the anatomical borders and increases the accuracy of the calcium quantification score. Previously, calcium score determination was solely based on non-contrast scans (with 3mm thick slices) in a manual application tool without multiplanar reconstructions and less accurate delineation of the region of interest. Figure 2 highlights the different calcium selection methods between 3mensio and IntelliSpace. This calcium module is a complementary tool to quantify aortic valve calcium burden and may help differentiate between moderate and severe AS in the context of borderline or low-flow, low-gradient AS, determine whether balloon predilatation is required and result in a patient-tailored transcatheter valve selection.

### Limitations

This study is a retrospective study with a relatively small sample size. Calcium quantification with the different analysis modalities was performed by experienced imagers, but not in a Core Laboratory setting. For this analysis, only patients with good quality CT studies were included. This validation study should therefore not be extrapolated to low quality CT scans.

## Conclusions

The semiautomated calcium quantification module in 3mensio Structural Heart highly correlated with a conventional manual calcium scoring tool.

## Ethics Statement

The study was conducted in accordance with the Declaration of Helsinki and did not fall under the scope of the Medical Research Involving Human Subjects Act per institutional review boards’ review.

## Funding

The authors have no funding to report.

## Disclosure Statement

Nicolas M. Van Mieghem received research grants and advisory fees from Abbott, Boston Scientific Corporation, Edwards Lifesciences, Medtronic, Teleflex, Daiichi Sankyo; and from Ancora Heart. Joost Daemen received institutional grant/research support from Astra Zeneca, Abbott Vascular, Boston Scientific, ACIST Medical, Medtronic, Pie Medical, and ReCor medical. The other authors had no conflicts to declare.
